# 5-Aminolaevulinic Acid (ALA) for the Fluorescence Detection of Bronchial Tumors

**DOI:** 10.1155/DTE.5.113

**Published:** 1999

**Authors:** Rudolf M. Huber, F. Gamarra, H. Hautmann, K. Häußinger, S. Wagner, M. Castro, R. Baumgartner

**Affiliations:** 1 Division of Pneumology Department of Medicine Klinikum Innenstadt, University of Munich Ziemssenstraße 1 München D-80336 Germany; 2 Hospital for Pneumology University of Munich Germany; 3 Laser Research Institute Department of Urology University of Munich Germany

## Abstract

At the moment only early detection of lung cancer offers a good prognosis for the patients. Conventional white light endoscopy is mostly insufficient for early diagnosis. Therefore we developed a system of fluorescence diagnosis using 5-aminolaevulinic acid (ALA) exogeneously applied. As precursor of the heme synthesis it is metabolized to protoporphyrin IX –  a red fluorescent substance. Therefore protoporphyrin IX accumulates in tumorous and premalignant tissue, and can be directly visualized by fluorescence bronchoscopy. Excitation with blue light (380–435 nm) causes a red fluorescence, which can be detected after filtering most of the blue component with the naked eye or a camera system. After earlier work with laser systems and cold light sources we now use the system D-Light AF for the fluorescence diagnosis using ALA-induced protoporphyrin IX fluorescence.


*Diagnostic and Therapeutic Endoscopy*, Vol. 5, pp. 113–118
Reprints available directly from the publisher
Photocopying permitted by license only

© 1999 OPA (Overseas Publishers Association) N.V.
Published by license under
the Harwood Academic Publishers imprint,
part of The Gordon and Breach Publishing Group.
Printed in Singapore.

### 5-Aminolaevulinic Acid (ALA) for the Fluorescence Detection of Bronchial Tumors

RUDOLF M. HUBER[a,*], F. GAMARRA[a], H. HAUTMANN[a], K. HÄUßINGER[b], S. WAGNER[c], M. CASTRO[c] and R. BAUMGARTNER[c]

[a]*Division of Pneumology, Department of Medicine, Klinikum Innenstadt, University of Munich, Ziemssenstraße 1, D-80336 München, Germany;* [b]*Hospital for Pneumology,* [c]*Laser Research Institute, Department of Urology, University of Munich, Germany*

**At the moment only early detection of lung cancer offers a good prognosis for the patients. Conventional white light endoscopy is mostly insufficient for early diagnosis. Therefore we developed a system of fluorescence diagnosis using 5-aminolaevulinic acid (ALA) exogeneously applied. As precursor of the heme synthesis it is metabolized to protoporphyrin IX – a red fluorescent substance. Therefore protoporphyrin IX accumulates in tumorous and premalignant tissue, and can be directly visualized by fluorescence bronchoscopy. Excitation with blue light (380–435 nm) causes a red fluorescence, which can be detected after filtering most of the blue component with the naked eye or a camera system. After earlier work with laser systems and cold light sources we now use the system D-Light AF for the fluorescence diagnosis using ALA-induced protoporphyrin IX fluorescence.**

*Keywords:* 5-Aminolaevulinic acid (ALA), Protoporphyrin IX, Bronchoscopy, D-Light AF, Early detection, Lung cancer

### INTRODUCTION

Lung cancer is a very common disease throughout the world, which has a dismal prognosis. The incidence of lung cancer is still rising in most parts of the world. Only early stages of lung cancer can be cured with a high probability [1]. Therefore tools for screening and early diagnosis are necessary [2]. For non-invasive screening X-ray of the thorax and sputum cytology were applied [3–7] and got inconclusive results. Therefore these methods are now reexamined [8]. In the meantime the computed tomography of the thorax is investigated [9] and improvements of the sputum cytology were developed like immune cytology using antibody staining and automated morphometrical analyses [10,11].

If there are suspicious results or the patient has a history suggesting a lung cancer, bronchoscopy is done. But bronchoscopy has a low sensitivity for early changes [12]. Therefore methods for improving the detection of early lesions are needed. Apart from autofluorescence systems (see [13,14] and

* Corresponding author. Tel.: +49 89 51602590. Fax: +49 89 51604905. E-mail: huber@medinn.med.uni-muenchen.de.

other articles in this issue) active fluorescence detection using photosensitizers is used. Early work was done with hematoporphyrin derivatives in therapeutic dosages [15–17], but these efforts were limited by the skin phototoxicity of these substances. Therefore more sophisticated methods using two-wavelength systems or time-gated systems were applied [18–20]. The introduction of 5-aminolaevulinic acid (5-ALA) as an endogeneous photosensitizer [21] has changed the field and allows a very simple system for photodynamic detection of early tumorous or premalignant lesions in various organs such as the bladder [22] and the brain [23].

### MATERIALS AND METHODS

### Mechanism of 5-Aminlaevulinic Acid Induced Protopophyrin IX Fluorescence

5-aminolaevulinic acid is a naturally occurring substance which is normally synthesized from succinyl Co A and glycin. This synthesis is negatively regulated by the presence of heme. If 5-ALA is applied exogeneously, this negative feed-back mechanism is surpassed and protoporphyrin IX is synthesized during the heme biosynthesis. Figure 1 demonstrates schematically the principles of the accumulation of protoporphyrin IX in the tumorous tissue.

The pharmacokinetics of the protoporphyrin IX synthesis and metabolism are much more suited to clinical applications than for, as an example, those of hematoporphyrin derivatives. After 24 h even with high systemic dosages of 5-ALA, there are no relevant, higher levels of protoporphyrin IX in the skin or the systemic circulation. After inhalation there are only very small levels detectable during a one day period [24,25]. The mechanisms for the relatively selective accumulation of protoporphyrin IX are not quite understood, but are at least partly caused by different enzyme activities between malignant and normal cells.

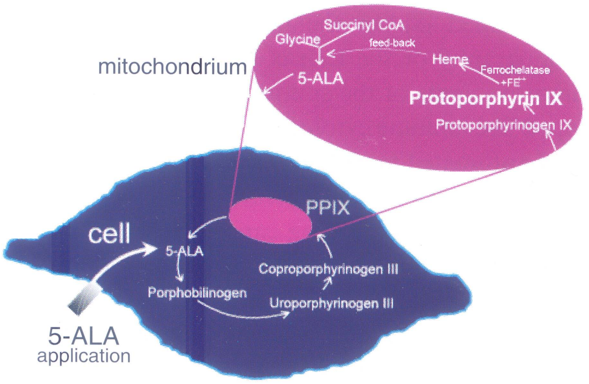

FIGURE 1 Demonstration of the principles of the accumulation of protoporphyrin IX in the tumorous tissue.

### Three-dimensional Cell Culture Systems

After the first reports of the use of 5-ALA on the skin and in the bladder we decided to examine the application of 5-ALA in the bronchial system. We examined the application of 5-ALA in three-dimensional miniorgans [26], which are derived from biopsies of the bronchial wall. For these experiments these biopsies are taken in routine bronchoscopies and cultivated for 14 days. We found that after a short exposition 5-ALA is metabolized in these cultures with normal epithelium to protoporphyrin IX. This process has its maximum between one and two hours [27].

We were further interested in whether there is a difference between the normal epithelium and tumorous tissue. For these purposes we established a three-dimensional coculture system between the above-described system and invading tumor cells [28]. We found after a short exposition of 15 min a clear difference between the normal epithelium and the tumor. The maximum difference between the normal and the tumorous tissue was between about 100 and 200 min [29].

Figure 2 demonstrates an example of a coculture with the enhanced red fluorescence in the tumorous areas in contrast to the normal epithelium.

### Animal Experiments

In animal experiments with beagle dogs with induced tumors, we found after nebulization of 5-ALA a clear and visible difference between the normal and tumorous areas.

As 5-ALA is an acid, we were concerned about the possibility of bronchoconstriction after inhalation of this substance. We tested this situation in beagle dogs and found no evidence for a bronchoconstriction through the nebulization which lasted over 10 min with 25% 5-ALA diluted in saline. Figure 3 demonstrates as an example of the lung function parameters the course of resistance.

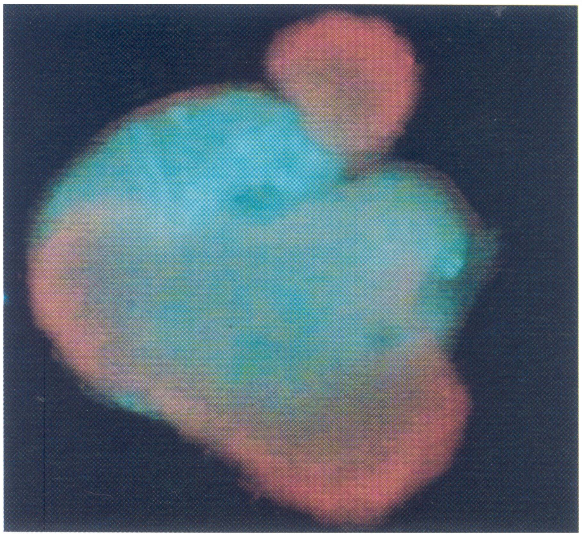

FIGURE 2 Example of a coculture with the enhanced red fluorescence in the tumorous areas in contrast to the normal epithelium.

### PRELIMINARY CLINICAL RESULTS

In more than 100 patients with an established or suspected lung cancer, we were able to demonstrate that the inhalation of 200 mg of 5-ALA (Company Medac GmbH, Hamburg, Germany) diluted in 5 ml NaCl 0.9% by a conventional jet nebulizer (Company Pari-Medanz, Starnberg, Germany) or a volume and flow controlled inhalation device for a homogenous deposition (GSF-Inhaler: Institute for Inhalation Biology – H. Schulz, P. Brand) with a dose of 60 mg in 2.5 ml NaCl 0.9% is feasible and safe. There are no side effects apart from the occasional cough in these mostly bronchitic patients. Figure 4 shows as an example the peak flow values of our first patients which demonstrates no deterioration of the individual values.

The results of our *in vitro* study regarding the best time interval between inhalation and photodynamic diagnosis are now being evaluated in patients in a Phase-I-study using the D-Light AF-system from Storz (in cooperation with the company Medac). In the same study we are evaluating the differences between the two types of inhalation (Klinikum Innenstadt: jet nebulization with the Pari-Inhaler; Hospital for Pneumology, München-Gauting: optimized deposition with the GSF-Inhaler). About 90–120 min after inhalation, we conduct bronchoscopy mostly with flexible endoscopes. Rigid endoscopes are used in combination with a camera and

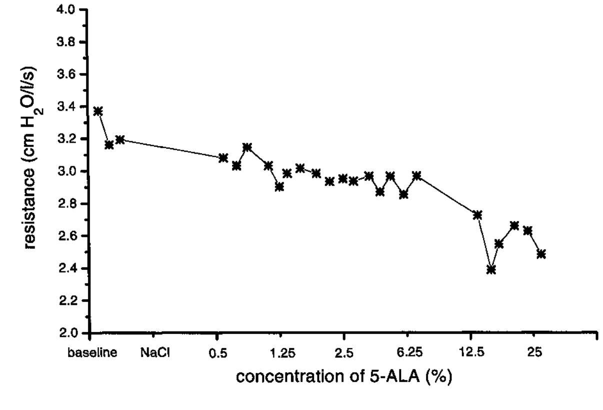

FIGURE 3 Course of airway resistance during nebulization of 5-ALA in a beagle dog.

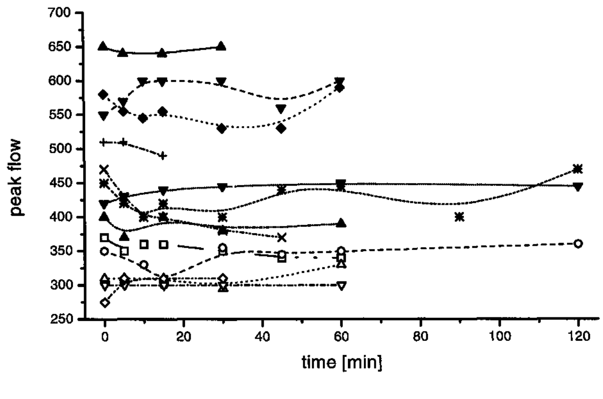

FIGURE 4 Peak flow values during and after inhalation of 5-ALA demonstrating no relevant effect on pulmonary function.

tangential illumination which is a little bit more non-specific.

As protoporphyrin IX can be bleached by too much light, we begin the examination with the ALA-mode. After this we perform a routine white light bronchoscopy. We take biopsies from all suspect regions on the white light bronchoscopy and the fluorescence bronchoscopy. Apart from these visible areas, random biopsies are also taken.

The method is very sensitive. Apart from big and necrotic tumors, all tumors, carcinomata *in situ*, and severe dysplastic areas show a positive red fluorescence. But there are also cases, in up to 50% of the circumscript areas of red fluorescence, which are only bronchitic or only mildly dysplastic. There are suggestions that these lesions are highly proliferating or genetically altered regions of the epithelium according to the findings in urology [30].

Since autofluorescene excitation ranges from 380 to 460 nm, fluorescence of protoporphyrin IX can be seen together with the autofluorescence. Our first experiments in patients show that the fluorescence diagnosis using protoporphyrin IX fluorescence *and* autofluorescence is possible and feasible. Whether we really get a better specificity using a combination with autofluorescence (AF-mode/AF-filters) is being tested in a pilot-study.

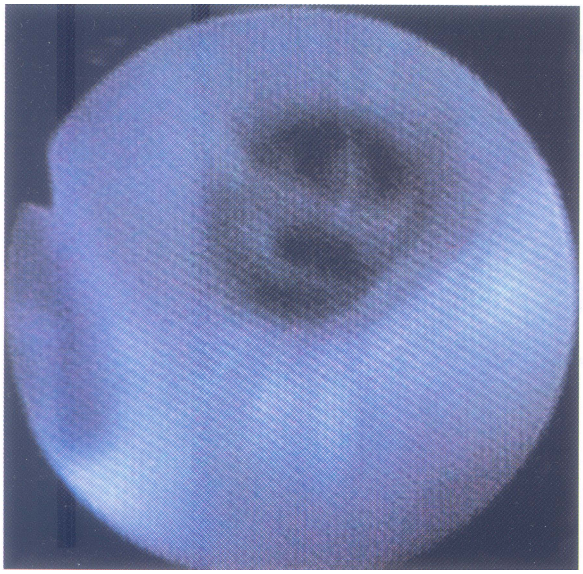

FIGURE 5 Bronchoscopic picture of a normal fluorescence of a bronchus.

Figure 5 demonstrates a normal fluorescence of a bronchus in a bronchoscopic picture. In Figure 6(a) and (b) the bronchoscopic pictures of a pathologic fluorescence and the corresponding white light bronchoscopy of a neoplastic lesion can be seen.

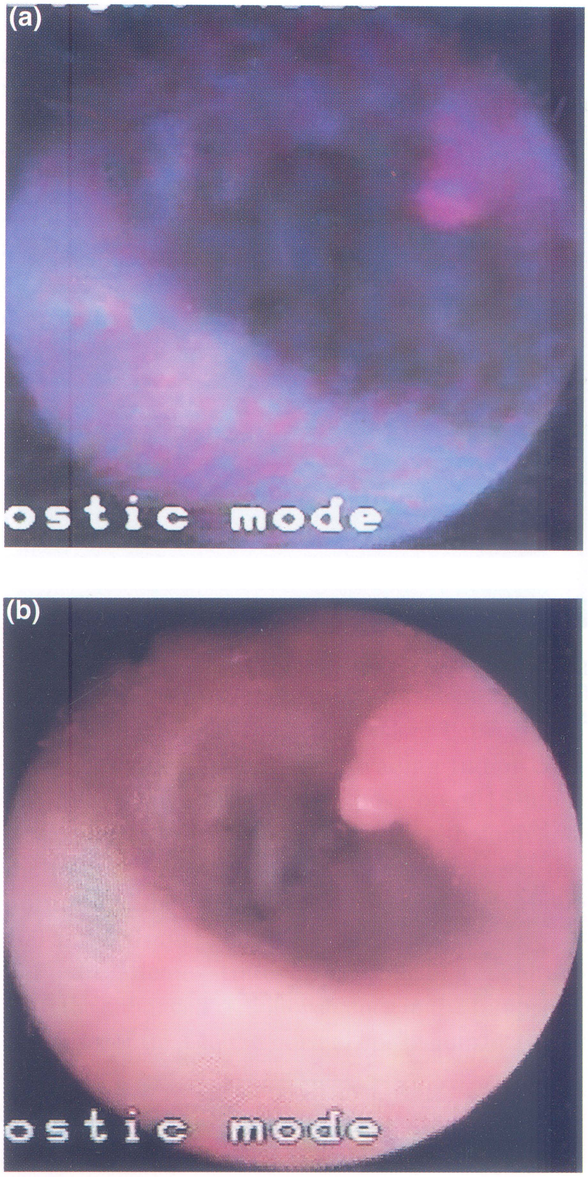

FIGURE 6 Bronchoscopic pictures of a pathologic fluorescence (a) and the corresponding white light bronchoscopy (b) of a neoplastic lesion.

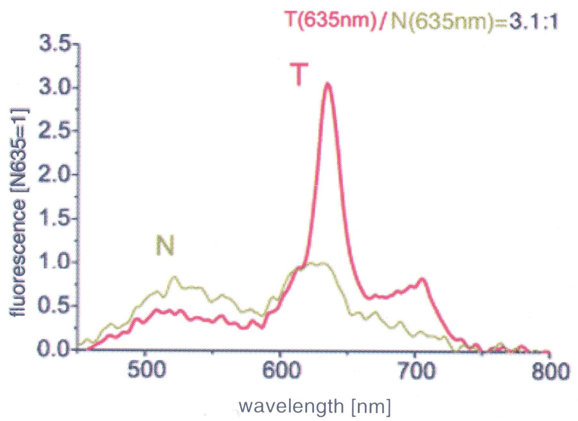

FIGURE 7 Spectral data of normal (green line) and severely dysplastic epithelium (red line). The ratio is about 3 : 1.

Figure 7 illustrates spectral data of a normal epithelium and a severe dysplasia. The ratio is about 3 : 1 for the severe dysplasia. The great difference and the good optical conditions result in a high contrast between the blue fluorescence of the normal tissue and the specific red fluorescence of tumorous tissue.

### DISCUSSION

Lung cancer is a very serious health problem. Only early therapy offers a chance for cure. For optimal therapy it is further very important to know the exact borders of the tumor and not to miss other endobronchial neoplastic areas. Apart from non-invasive screening methods we need therefore better bronchoscopic techniques. For example the conventional white light bronchoscopy misses carcinomata *in situ* in up to 70% of the cases [12]. Earlier solutions using pharmacological fluorescence of tumorous areas were very complicated and expensive. Now we have two methods applicable in the daily routine: the autofluorescence and the fluorescence of protoporphyrin IX induced by exogeneously applied 5-ALA. 5-aminolaevulinic acid can be applied by simple inhalation, but of course can also be applied systemically, if there is photodynamic therapy with 5-ALA planned. A relative disadvantage of the protoporphyrin fluorescence induced by exogeneously applied ALA is that the bronchoscopy can only be done some time (about 90–120 min) after the inhalation of 5-ALA. The effects of autofluorescence are mostly determined from the tissue and the morphological changes, whereas protoporphyrin IX-fluorescence is further determined by metabolic processes and probably more tumor dependent/specific. So there should be differences between the two methods. A combination of the two methods could enhance the specificity of tumor detection by fluorescence methods. The 5-ALA-induced fluorescence is further useful for the planning of photodynamic therapy, as it can demonstrate the area which should be illuminated. We believe that there should be a prospective randomized comparison between the two methods. Further the combination of autofluorescence and protoporphyrin fluorescence, induced by ALA should be thoroughly evaluated.

### *Acknowledgements*

We kindly thank our industrial partners at Karl Storz GmbH & Co. in Tuttlingen, Germany, for their support and brilliant technical work.

### *References*

[1] Cortese, D.A., Pairolero, P.C., Bergstrath, E.J., Woolner, L.B., Uhlenhopp, M.A., Piehler, J.M., Sanderson, D.R., Bernatz, P.E., Williams, D.E., Taylor, W.F., Payne, W.S., Fontana, R.S. Roentgenographically occult lung cancer. A ten-year experience. *J. Thorac. Cardiovasc. Surgery* 1983; **86**: 373–380.

[2] Wagner, H. Jr. and Ruckdeschel, J.C. Lung cancer in: Reintgen D.S. and Clark R.A. (eds.). *Cancer Screening.* Mosby. St. Louis 1996.

[3] Fontana, R.S., Sanderson, D.R., Woolner, L.B., Taylor, W.F., Miller, W.E., Muhm, J.R., Bernatz, P.E., Payne, W.S., Pairolero, P.C. and Bergstralh, E.J. Screening for lung cancer. A critique of the Mayo Lung Project. *Cancer* 1991; **67**: 1155–1164.

[4] Fontana, R.S., Sanderson, D.R., Taylor, W.F., Woolner, L.B., Miller, W.E., Muhm, J.R. and Uhlenhopp, M.A. Early lung cancer detection: results of the initial (prevalence) radiologic and cytologic screening in the Mayo Clinic study. *Am. Rev. Respir. Dis.* 1984; **130**: 561–565.

[5] Frost, J.K., Ball, W.C. Jr., Levin, M.L., Tockman, M.S., Baker, R.R., Carter, D., Eggleston, J.C., Erozan, Y.S., Gupta, P.K., Khouri, N.F. *et al.* Early lung cancer detection: results of the initial (prevalence) radiologic and cytologic screening in the Johns Hopkins study. *Am. Rev. Respir. Dis.* 1984; **130**: 549–554.

[6] Melamed, M.R., Flehinger, B.J., Zaman, M.B., Heelan, R.T., Perchick, W.A., and Martini, N. Screening for early lung cancer. Results of the Memorial Sloan-Kettering study in New York. *Chest* 1984; **86**: 44–53.

[7] Kubik, A. and Polak, J. Lung cancer detection. Results of a randomized prospective study in Czechoslovakia. *Cancer* 1986; **57**: 2427–2437.

[8] Strauss, G.M., Gleason, R.E. and Sugarbaker, D.J. Screening for lung cancer. Another look; a different view *Chest* 1997; **111**: 754–768.

[9] Mori, K., Tominaga, K., Hirose, T., Sasagawa, M., Yokoyama, K. and Moriyama, N. Utility of low-dose helical CT as a second step after plain chest radiography for mass screening for lung cancer. *J. Thorac. Imaging* 1997; **12**: 173–180.

[10] Tockman, M.S. and Mulshine, J.L. Sputum screening by quantitative microscopy: A new dawn for detection of lung cancer? *Mayo Clin. Proc.* 1997; **72**: 788–790.

[11] Payne, P.W., Sebo, T.J., Doudkine, A., Garner, D., MacAulay, C., Lam, S., LeRiche, J.C. and Palcic, B. Sputum screening by quantitative microscopy: A reexamination of a portion of the National Cancer Institute Cooperative Early Lung Cancer Study. *Mayo Clin. Proc.* 1997; **72**: 697–704.

[12] Woolner, L.B., Fontana, R.S., Cortese, D.A., Sanderson, D.R., Bernatz, P.E., Payne, W.S., Pairolero, P.C., Piehler, J.M. and Taylor, W.F. Roentgenographically occult lung cancer: pathologic findings and frequency of multicentricity during a 10-year period. *Mayo Clin. Proc.* 1984; **59**: 453–466.

[13] Hung, J., Lam, S., Jaggi, B., Eng, P., Pon, A., and Balcic, B. Localization of lung cancer by tissue autofluorescence. *Chest* 1990; **98**(Suppl): 108S.

[14] Lam, S., Kennedy, T., Unger, M., Miller, Y.E., Gelmont, D., Rusch, V., Gipe, B., Howard, D., LeRiche, J.C., Coldman, A. and Gazdar, A.F. Localization of bronchial intraepithelial neoplastic lesions by fluorescence bronchoscopy. *Chest* 1998; **113**: 696–702.

[15] Cortese, D.A., Kinsey, J.H., Woolner, L.B., Payne, W.S., Sanderson, D.R. and Fontana, R.S. Clinical application of a new endoscopic technique for detection of *in situ* bronchial carcinoma. *Mayo Clin. Proc.* 1979; **54**: 635–641.

[16] Hayata, Y., Kato, H., Ono, J., Matsushima, Y., Hayashi, N., Saito, T. and Kawate, K. Fluorescence fiberoptic Bronchoscopy in the diagnosis of early stage lung cancer. Recent Results. *Cancer Res.* 1982; **82**: 121–130.

[17] Kato, H. and Cortese, D.A. Early detection of lung cancer by means of hematoporphyrin derivative fluorescence and laser photoradiation. *Clin. Chest. Med.* 1985; **6**: 237–253.

[18] Kato, H., Aizawa, K., Ono, J., Konaka, C., Kawate, N., Yoneyama, K., Kinoshita, K., Nishimiya, K., Sakai, H., Noguchi, M., Tomono, T., Kawasaki, S., Tokuda, Y. and Hayata, Y. Clinical measurement of fluorescence using a new diagnostic system with hematoporphyrin derivative, laser photoradiation, and a spectroscope. *Lasers Surg. Med.* 1984; **4**: 49–58.

[19] Edell, E.S. and Cortese, D.A. Bronchoscopic localization and treatment of occult lung cancer. *Chest* 1989; **96**: 919–921.

[20] Baumgartner, R. and Unsöld, E. High contrast fluorescence imaging using two-wavelength laser excitation and image processing. *J. Photochem. Photobiol. B* 1987; **1**: 130–132.

[21] Kennedy, J.C. and Pottier, R.H. Endogenous protoporphyrin IX, a clinically useful photosensitizer for photodynamic therapy. *J. Photochem. Photobiol. B* 1992; **14**: 275–292.

[22] Kriegmair, M., Baumgartner, R., Knüchel, R., Stepp, H., Hofstaedter, F. and Hofstetter, A. Detection of early bladder cancer by 5-aminolevulinic acid induced porphyrin fluorescence (see comments) *J. Urol.* 1996; **155**: 105–109; discussion 109–110.

[23] Stummer, W., Stocker, S., Wagner, S., Stepp, H., Fritsch, C., Goetz, C., Goetz, A.E., Kiefmann, R. and Reulen, H.J. Intraoperative detection of malignant gliomas by 5-aminolevulinic acid-induced porphyrin fluorescence. *Neurosurgery* 1998; **42**: 518–525; discussion 525–526.

[24] Baumgartner, R., Huber, R.M., Schulz, H., Stepp, H., Rick, K., Gamarra, F., Leberig, A. and Roth, C. Inhalation of 5-aminolevulinic acid: A new techique for fluorescence detection of early stage lung cancer. *J. Photochem. Photobiol. B* 1996; **36**: 169–174.

[25] Rick, K., Sroka, R., Stepp, H., Kriegmair, M., Huber, R.M., Jacob, K. and Baumgartner, R. Pharmacokinetics of 5-aminolevulinic acid-induced protoporphyrin IX in skin and blood. *J. Photochem. Photobiol. B* 1997; **40**: 313–319.

[26] Bals, R., Gamarra, F., Kaps, A., Grundler, S., Huber, R.M. and Welsch, U. Secretory cell types and cell proliferation of human bronchial epithelial cells in an organ-culture system. *Cell and Tissue Research* 1998; **293**: 573–577.

[27] Maier, I., Stepp, H., Huber, R.M., Rick, K., Baumgartner, R. and Gamarra, F. Kinetics of protoporphyrin-IX fluorescence after incubation with 5-Aminolaevulinic Acid in organ cultures of human bronchial epithelium. *Eur. Respir. J.* 1996; **9**: 187s.

[28] Al-Batran, S., Gamarra, F., Astner, S., Knüchel, R. and Huber, R.M. Three-dimensional *in vitro* cocultivation of lung carcinoma cells with human bronchial organ cultures. Submitted.

[29] Huber, R.M., Gamarra, F., Castro, M., Wagner, S., Maier, I., Stepp, H. and Baumgartner, R. Kinetics of 5-aminolaevulinic aca (5-ALA) induced protoporphyrin IX (PPIX) fluorescence in tumor infiltrated organ cultures of human bronchial epithelium. *Proceedings Congress International Photodynamic Association* 1998 (in press).

[30] Zaak, D., Stepp, H., Baumgartner, R., Knüchel, R., Kriegmair, R. and Hofstetter, A. Endoscopic detection of urinary bladder cancer with 5-ALA induced fluorescence. 7th Biennial Congress International Photodynamic Association. Nantes Book of Abstracts 1998.

